# 

**Published:** 1881-07

**Authors:** 


### *American Dental Association.*

The next Annual Meeting of this Association will be held in the city of New York, on Thursday July 12, 1881. This change of time has been decided upon by the Executive Committee and the Officers of the Association, in view of the fact that some forty or fifty of the members will probably be absent at the international congress, to be held in London on the 2d–9th of August.

Circulars will be sent to all members of the Association, with further particulars.

C. N. Pierce, *Pres't.*

Geo. H. Cushing, *Sec.*

The local Committee of Arrangements would recommend the Westminister Hotel, corner of Sixteenth Street and Irving Place, as a very quiet and good hotel, and as being in close proximity to the place of meeting. They have reduced the price from their regular rates of $3.50 to $2.50 per day.

If any members desire to secure rooms and will write to me, I will secure the best I can.

A. L. Northrop,
*Chr'n. Com. of Arrangements.*

44 West Forty-sixth Street, New York City.

---

*National Dental Association.*

The regular annual meeting of the Dental Association of the United States of America will be held in New York City commencing Monday, August 8th, 1881.

This Association was organized, not in opposition to those already existing, but to supply a want that is not met by any one of them. It is so constituted that members of the Profession in all parts of the Country can in turn attend the meetings and participate in its control and its benefits.

All members of the Profession are therefore cordially invited to attend this meeting.

R. Finley Hunt, *Sec'y.*,

Washington, D. C.

---

*Southern and North Carolina Dental Associations.*

The Thirteenth Annual Meeting of the Southern Dental Association will take place at Ashville, North Carolina, on the last Tuesday in July (26th,) 1881.

The North Carolina Dental Association meets at the same time and place.

Every arrangement that will conduce to the pleasure of the occasion has and will be made.

All Dentists are invited to be present at this delightful place, and have a good time.

E. S. Chisholm, *Rec. Sec. S. D. A.*

*Alabama Dental Association.*

The next Annual Meeting of the Alabama Dental Association will be held in Selma, Ala., on the third Tuesday (19th day) of July, 1881.

The State Board of Dental Examiners will meet at same time and place. Exery Dentist practicing in the State is expected to be present, as under the late act of the Legislature, every one practicing dentistry in the State is *compelled to have* a license from this Board.

T. M. Allen, *Rec. Sec., Eufaula, Ala.*

---

*American Dental Convention.*

Owing to the fact of a large number of dentists intending to visit Europe during the session of the Medical and Dental Convention in London, it has been thought advisable to defer the meeting of the above convention until later. Due notice of time and place will be given.

John Allen, *Pres't.*

J. G. Ambler, *Ch'n. Com. of Arrangements.*

---

### EDITORIAL, ETC.

### To Readers and Correspondents.

Communications intended for publication, and exchange journals and papers, should be addressed to the EDITOR OF THE AMERICAN JOURNAL OF DENTAL SCIENCE, 259 N. Eutaw St., Baltimore, Md. All letters relating to the business of the journal, or containing cash remittances, to be directed to Messrs. SNOWDEN & COWMAN. Articles for publication should be received by the editor at least three weeks previous to the first of the month on which the number in which it is designed for them to appear, is issued.

### EDITORIAL, ETC.

The Bonwill Electro-Magnetic Mallet.................135
Another Journal.................137
Endorsements.................139
Preparatory Education.................139
Therapy.................140

### MONTHLY SUMMARY.

The Preservation of the Teeth.................141
Food for Infants.................141
Salivary Calculus.................142
Apparent Insusceptibility to the Influence of Nitrous Oxide.................143
Peculiarities of Race.................144
Peculiar Obstruction of Wharton's Duct.................144

☞ The American Journal of Dental Science will contain fifty pages of reading matter in each number, making a volume of about *Six Hundred* pages,

EXCLUSIVE OF ADVERTISEMENTS.

THE

### AMERICAN JOURNAL

OF

### DENTAL SCIENCE.

The *Journal* is issued on the first of each month and contains not less than *fifty* pages of reading matter in each number, exclusive of advertisements, making a volume of *six hundred* pages per annum.

In each number will be found Original Communications, Correspondence, Selected Articles, Monthly Summary, Bibliographical Notices and Editorial Matter, and every effort will be exerted to render the *Journal* worthy of the patronage of the Dental profession.

A generous co-operation is respectfully solicited from the members of the profession; and as the *Journal* has now a printing office of its own, which materially lessens the cost of its publication, it will be issued at the reduced subscription price of

**$2.50 Per Annum in Advance.**

Communications are respectfully solicited on all subjects pertaining to the practice of dentistry, as well as reports of cases occurring in dental practice.

### RATES OF ADVERTISING.

| | 1 MO. | 2 MO. | 3 MO. | 6 MO. | 12 MO. |
|---|---|---|---|---|---|
| 1 PAGE. | $15 00 | $22 50 | $30 00 | $50 00 | $100 00 |
| ½ " | 10 00 | 15 00 | 20 00 | 30 00 | 50 00 |
| ¼ " | 7 00 | 11 00 | 15 00 | 20 00 | 30 00 |
| ⅛ " | 5 00 | 8 00 | 11 00 | 15 00 | 20 00 |

All original communications designed for publication, works for review, new instruments for notice, exchanges, &c., should be directed to the editor, 259 N. Eutaw St., Baltimore, Md.

All advertisements and subscriptions should be addressed to

SNOWDEN & COWMAN,

*Publishers of the Am. Journal of Dental Science.*

86 W. FAYETTE ST. BET. CHARLES & LIBERTY STS.,

BALTIMORE, MD.

